# Base editing: a promising tool to rescue spinal muscular atrophy

**DOI:** 10.1038/s41392-023-01583-5

**Published:** 2023-09-25

**Authors:** Priyanka Bhatia, Jared Sterneckert

**Affiliations:** 1 Center for Regenerative Therapies TU Dresden (CRTD), Technische Universität (TU) Dresden, Dresden, Germany; 2 Medical Faculty Carl Gustav Carus of TU Dresden, Dresden, Germany

**Keywords:** Diseases of the nervous system, Gene therapy

In a new *Science* publication, Arbab and colleagues observe a substantial improvement in life span and motor functions of adenosine-base edited spinal muscular atrophy (SMA) mice with restored endogenous survival motor neuron (SMN) protein expression and regulation.^1^ This study provides proof-of-principle for the use of adenosine base editing (ABE) as a possible one-time, permanent therapy for SMA.

SMA is a motor neurodegenerative disease. In its most common form (type I), symptoms manifest at birth, and patients survive a median of six months. SMA is caused due to homozygous deletion or mutation of *SMN1*, leading to loss of SMN protein, resulting in the death of motor neurons. Depending on the number of copies, protein from *SMN2* can compensate for *SMN1* loss. However, a single silent C to T mutation in position 6 of exon 7 (C6T) causes a change in splicing. About 90% of the mRNAs transcribed from *SMN2* do not include exon 7, resulting in a truncated protein (SMNΔ7) with an aberrant EMLA degron that is quickly degraded.^2^ Only about 10% of mRNAs transcribed by *SMN2* encode full-length protein.

Three therapeutics are approved for SMA, all of which aim to increase the levels of SMN protein. Nusinersen (an antisense-oligonucleotide) and Risdiplam (a small molecule) alter the splicing of *SMN2*. However, these treatments are transient, requiring repeated dosing for life, and may not facilitate the complete rescue of protein levels. Zolgensma is a gene therapeutic leading to the production of SMN protein, but not under the control of the endogenous promoter, leading to incomplete restoration of SMN transcript levels in the spinal cord and expression in other tissues that could lead to toxicity.^3^ Thus, a pivotal therapeutic goal for SMA patients is to achieve permanent and endogenous regulation of rescued SMN levels.

**Fig. 1:**
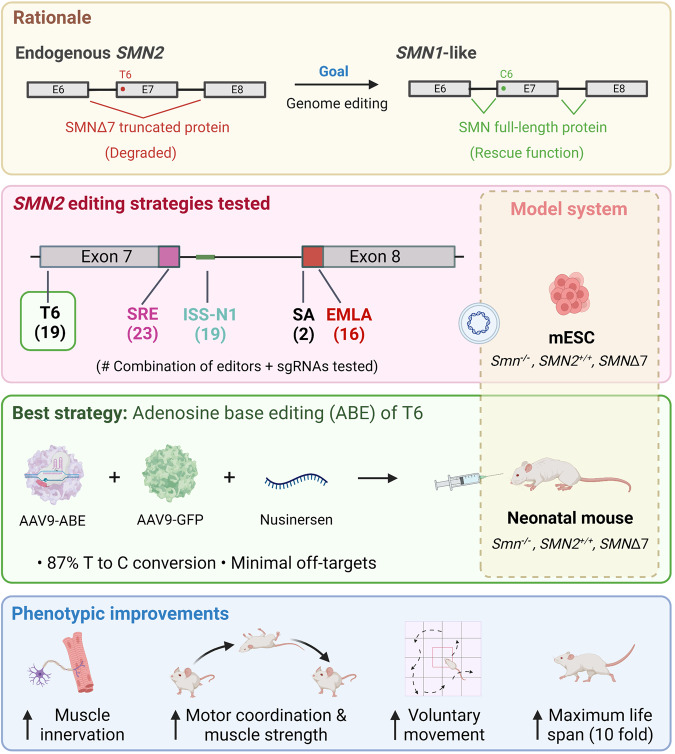
Schematic overview of the experiments by Arbab and colleagues in which genome-editing technology was used to modify endogenous *SMN2*. A T6 mutation in exon 7 results in deletion of exon 7 and premature truncation of the protein, which is quickly degraded. 5 different editing strategies were tested in mESCs to correct this defect, including targeting the T6, the exon 7 3’ splice regulatory elements (SRE), the intronic splicing silencer N1 (ISS-N1), the splice acceptor (SA) site of exon 8 and the EMLA degron. Adenosine base editing (ABE) of T6 > C was the most successful strategy, and AAV9 was used for delivery for in vivo testing in combination with AAV9-GFP as an infection control as well as Nusinersen, which successfully rescued SMA pathophysiology and resulted in behavioral improvements in SMA mice. Created with BioRender.com

Arbab and colleagues performed extensive testing of five different strategies to identify the most effective approach for increasing SMN protein levels (Fig. 1). As an initial model system, they used mouse embryonic stem cells (mESCs) that were modified to resemble the genetics of SMA patients (*Smn1*^*−/−*^ and carrying human *SMN2*^*+/+*^ and *SMNΔ7*). The first strategy disrupted the intronic splicing silencer N1 (ISS-N1) locus to improve exon 7 inclusion. Two (out of 19) combinations of SpCas9 nuclease variants and small guide RNAs (sgRNA), with high indel frequencies, successfully increased full-length SMN levels by 15-fold over untransfected cells.

Next, they disrupted the EMLA degron, thereby reducing the degradation of SMNΔ7 protein and increasing overall SMN protein levels.^4^ Interestingly, they observed that one editing combination (out of 16) produced higher levels of the modified SMNΔ7 protein, even though it had a lower precision of deletion.

Third, the splice acceptor (SA) site of exon 8 was targeted to improve SMN stability by comparing the efficiency of nuclease-based gene editing (C-nuc) to that of precise cytosine base editing (CBE).^5^ CBE significantly enhanced SMN levels to the same level as Risdiplam. Alternative splicing also produced transcripts that included exon 7, indicating that the increase in protein levels was due to the contributing full-length SMN protein.

Capitalizing on the accuracy and efficiency of the current repertoire of base editors, Arbab and colleagues focused on targeting single nucleotide substitutions that distinguish *SMN2* from *SMN1* and regulate splicing. The 3’ splice regulatory elements (SRE) of exon 7 (T44C, G52A, and A54G) were targeted using ABE8e, ABE7.10, and EA-BE4 deaminases with different guide RNAs and compatible SpCas9 variants, some of which improved exon 7 inclusion, but did not improve SMN protein levels as expected, perhaps due to additional imprecise missense changes.

The most effective strategy targeted the single nucleotide substitution C6T using the ABE8e base editor. Out of 19 tested combinations, one yielded a nearly complete T6 > C conversion, did not induce other indels, and led to few missense substitutions. This approach increased exon 7 inclusion and full-length SMN protein levels, even exceeding levels induced by Nusinersen and Risdiplam. Arbab and colleagues selected this candidate for in vivo investigation. Adeno-associated virus serotype 9 (AAV9) was used to deliver ABE8e-SpyMac and sgRNA (AAV9-ABE) in combination with AAV9-GFP (as a transduction control) to spinal cord motor neurons via intracerebroventicular (ICV) injection into Δ7SMA neonatal mice (*Smn*^*−/−*^ and carrying human *SMN2*^*+/+*^ and *SMNΔ7*). Successfully transduced GFP-positive cortical cells had approximately 87% T6 > C conversion with minimal undesired by-products over 18 weeks. These mice also showed an increased number and output of motor units innervating muscle fibers, indicating rescue of SMA pathophysiology. In addition, lifespan increased by 33% compared to untreated mice. The improvement in lifespan is highly dependent upon how early before symptom onset the treatment is administered and how long it takes for corrected protein expression, which determines the earliest possible time point for functional SMN protein to be available for motor neurons.

In an attempt to widen the therapeutic window before the onset of irreversible SMA damage and to better inform any potential clinical applications of this approach, the investigators co-administered Nusinersen with the AAV9-ABE and AAV9-GFP in Δ7SMA neonatal mice. Animals treated with Nusinersen in combination with AAV9-ABE showed improved motor coordination, muscle strength, voluntary movement, and weight compared with Nusinersen alone. The most striking result was the increase in life span, which more than tripled from an average of 28 days with only Nusinersen to an average of 111 days with Nusinersen and AAV9-ABE, and a subset of animals showed an increase in survival of over 10-fold.

When moving into clinical testing, the minimal dosage, optimal treatment window as well as potential side effects of extended base editor exposure will need to be addressed. Given the liver-associated toxicity reported in some instances of AAV9-based gene therapy, Zolgensma, similar off-target transduction concerns apply to the described AAV9 base editing approach. Additionally, given that humans can have multiple copies of *SMN2*, which impacts the clinical phenotypes manifested by SMA patients, an interesting challenge would be the possibility and consequence of efficiently editing more than one copy of *SMN2*. Overall, this study marks an important advance toward a one-time treatment for SMA and may break ground in the therapeutic application of base editing technologies in diseases with potential positive modifiers.
